# Human–Machine Interaction via Dual Modes of Voice and Gesture Enabled by Triboelectric Nanogenerator and Machine Learning

**DOI:** 10.1021/acsami.3c00566

**Published:** 2023-03-22

**Authors:** Hao Luo, Jingyi Du, Peng Yang, Yuxiang Shi, Zhaoqi Liu, Dehong Yang, Li Zheng, Xiangyu Chen, Zhong Lin Wang

**Affiliations:** † College of Mathematics and Physics, Shanghai Key Laboratory of Materials Protection and Advanced Materials in Electric Power, Shanghai University of Electric Power, Shanghai 200090, China; ‡ Beijing Key Laboratory of Micro-nano Energy and Sensor, Beijing Institute of Nanoenergy and Nanosystems, Chinese Academy of Sciences, Beijing 100083, PR China; § School of Nanoscience and Technology, University of Chinese Academy of Sciences, Beijing 100049, PR China

**Keywords:** triboelectric translator, silk protein, voice and gesture recognition, machine learning, human−machine interface

## Abstract

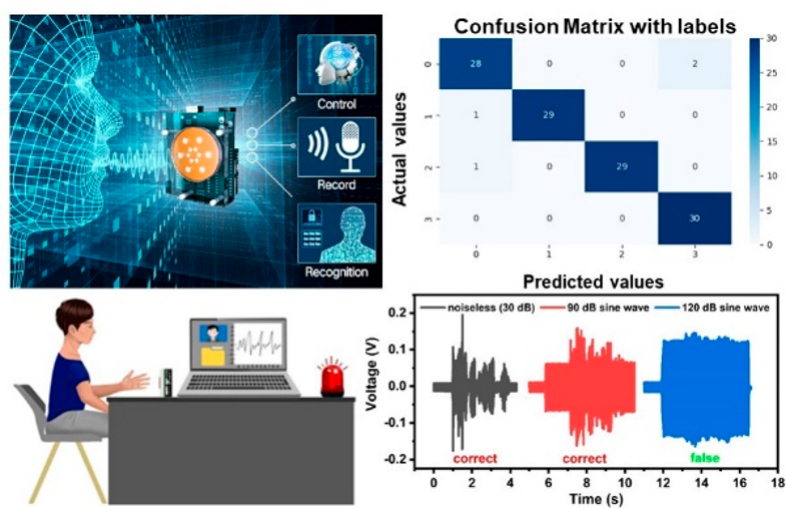

With the development of science and technology, human–machine interaction has brought great benefits to the society. Here, we design a voice and gesture signal translator (VGST), which can translate natural actions into electrical signals and realize efficient communication in human–machine interface. By spraying silk protein on the copper of the device, the VGST can achieve improved output and a wide frequency response of 20–2000 Hz with a high sensitivity of 167 mV/dB, and the resolution of frequency detection can reach 0.1 Hz. By designing its internal structure, its resonant frequency and output voltage can be adjusted. The VGST can be used as a high-fidelity platform to effectively recover recorded music and can also be combined with machine learning algorithms to realize the function of speech recognition with a high accuracy rate of 97%. It also has good antinoise performance to recognize speech correctly even in noisy environments. Meanwhile, in gesture recognition, the triboelectric translator is able to recognize simple hand gestures and to judge the distance between hand and the VGST based on the principle of electrostatic induction. This work demonstrates that triboelectric nanogenerator (TENG) technology can have great application prospects and significant advantages in human–machine interaction and high-fidelity platforms.

## 1 Introduction

Nowadays, the rapid development of artificial intelligence and the Internet of Things (IoT) has brought great changes to people’s lives, and human–machine interaction sets application requirements and research ideas for artificial intelligence and the Internet of Things (IoT).^1−7^ The technology of how to improve the efficiency of communication between human and machine is the most difficult problem for the researchers to overcome, in which the communication system^8−15^ connecting human and machine is particularly important. Nowadays, there are two main methods of human–machine interaction: voice–machine interaction^16−19^ and gesture–machine interaction.^20−23^ Voice signals contain rich character information on humans and are easy to use, so people can use voice signals to make and realize accurate commands. At present, the main sensors currently used for speech recognition are capacitive sensors,^24,25^ piezoresistive sensors,^26,27^ piezoelectric sensors,^28−30^ and triboelectric sensors.^31−33^ However, some components in capacitive and piezoresistive sensors, such as conventional commercial microphones, rely on battery charging and are vulnerable to environmental noise. On the other hand, piezoelectric sensors have complex structures, troublesome manufacturing process and short working frequency ranges. Fortunately, triboelectric nanogenerators (TENGs) can effectively solve these problems. In 2012, Wang et al. proposed the concept of triboelectric nanogenerator which is based on the coupling of contact electrification and electrostatic induction to collect various mechanical energy from the environment and convert it into electrical energy.^34−41^ Triboelectric nanogenerators have the advantage of low production cost, simple structure, high sensitivity and no external power supply. TENGs are quite stable in speech recognition and have a good broadband effect. It can respond sensitively to human voice in the frequency range of 20–2000 Hz. Some TENG-based acoustic sensors have been reported to use electrospinning to prepare porous elastic nanofiber membranes^42^ to collect sound signals using contact-separation mode. Although the membrane is thin and flexible and has good air permeability, the spinning process is cumbersome and many preparation materials are toxic. The final prepared membrane is not stable for long time use and a lot of information in frequency domain is missing. In 2018, Guo et al. invented a porous gold film TENG which has a broadband response,^43^ but it has low output, not high enough sensitivity, and the gold film is expensive. Therefore, it is worth investigating to prepare an acoustic TENG with good performance, simple to fabricate and eco-friendly.^44^

For gesture recognition, now the general recognition methods are mainly based on visual image analysis, but they are easy to be affected by light and occlusion. TENG can use electrostatic signal detection technology to sense the change of electrostatic field around the object so as to detect and recognize the target.^45,46^ It has the advantages of a small working blind area, simple system deployment, noncontact, and so on. However, a hybrid sensor for recognizing both voice and gesture sensor has never been realized by TENG technique.

In this paper, a voice and gesture signal translator (VGST) based on TENGs is designed and demonstrated for the first time. By spraying silk protein on the copper of the device, the VGST can be highly sensitive and has a wide range of frequency response (human ear hearing range 20–2000 Hz) with a higher sensitivity of 167 mV/dB. In comparison with previously reported acoustic sensors,^43^ the sensitivity of our device is improved by 51.8%, due to the superior performance of the silk protein. It also has strong anti-interference ability and high accuracy in recovering music. In addition, the VGST can be used for voice and gesture recognition through machine learning algorithms,^47^ and the recognition accuracy can reach 97%. In gesture recognition, four gestures can be clearly distinguished, and the distance from hand to device can be judged easily. This work demonstrates powerful functions of TENGs used in human–machine interfaces.

## 2 Results and Discussions

**Figure 1:**
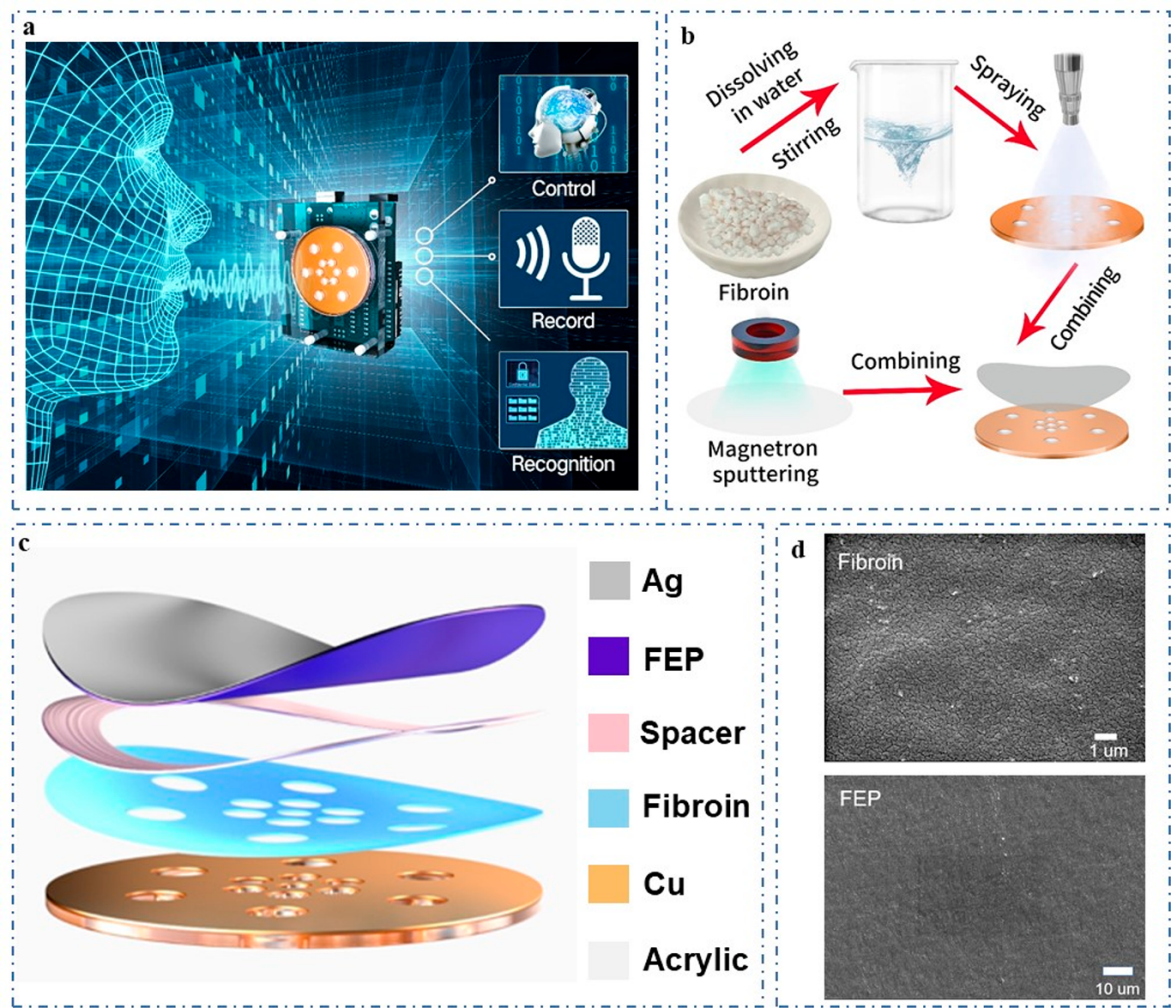
Application of the VGST in human–machine interaction and its structure. (a) Applications of the VGST in human–machine interaction. (b) Fabrication process of the VGST device consisting of FEP, Ag, fibroin, and Cu. (c) 3D decomposition structure of the VGST. (d) Scanning electron microscope image of silk protein and FEP surface.

TENG plays a very important role in many aspects of today’s big data era. Figure 1a demonstrates the ability of a TENG-based translator to efficiently receive acoustic signals and accurately respond. It has a wide range of application prospects in machine control, sound recognition, and recovery. The fabricated VGST could complete information management and enhance information security for people to the greatest possible extent, and could also help people with hearing impairments to better recover their hearing. Figure 1b illustrates the process of VGST fabrication. The specific process is described in the Experimental Section. The front and side view of the entire device is shown in Figure S1, with dimensions of 52.5 mm × 67.5 mm × 20 mm. The detailed three-dimensional decomposition structure of the VGST is shown in Figure 1c. The entire membrane part of VGST consists of 80 nm of silver, 12.5 um of FEP, 0.3 mm of spacer, 60 μm of copper and fibroin, and 2 mm of acrylic, showing that the device is very thin. Holes in the surface of the copper and fibroin are used to reduce air damping and to allow sound waves to better drive vibration of the membrane. To obtain the best sound detection, the size of the holes on the substrate is measured. If the holes are too small, then it is difficult to transmit the sound, thus the sound will be small and fine. If the holes are too large, then the high frequency sound will be sharp and harsh. Through lots of experiments, we found that it can obtain the best sound quality with radius of the outer holes of 3.2 mm and radius of the inner holes of 2 mm. Thus, weak sound of high frequency can also be accurately detected. We polarized the FEP film with a polarizer and injected charges to increase the surface charge density and then improve the electrification performance of FEP film. The picture of perforated copper sheet coated with silk protein is shown in Figure S2. The microstructure of FEP and fibroin is shown in Figure 1d. As can be seen from the SEM images, the surface of the FEP and fibroin is very smooth. As natural material with excellent physical and chemical properties, we sprayed silk protein on Cu film so as to fabricate sensitive VGST devices. Meanwhile, fibroin has high mechanical strength, lightweight and easy processing, and its strong ability to lose electrons can improve the output performance of the VGST. In addition, fibroin is nontoxic, harmless, and biocompatible, making it a good choice for triboelectric materials.

**Figure 2:**
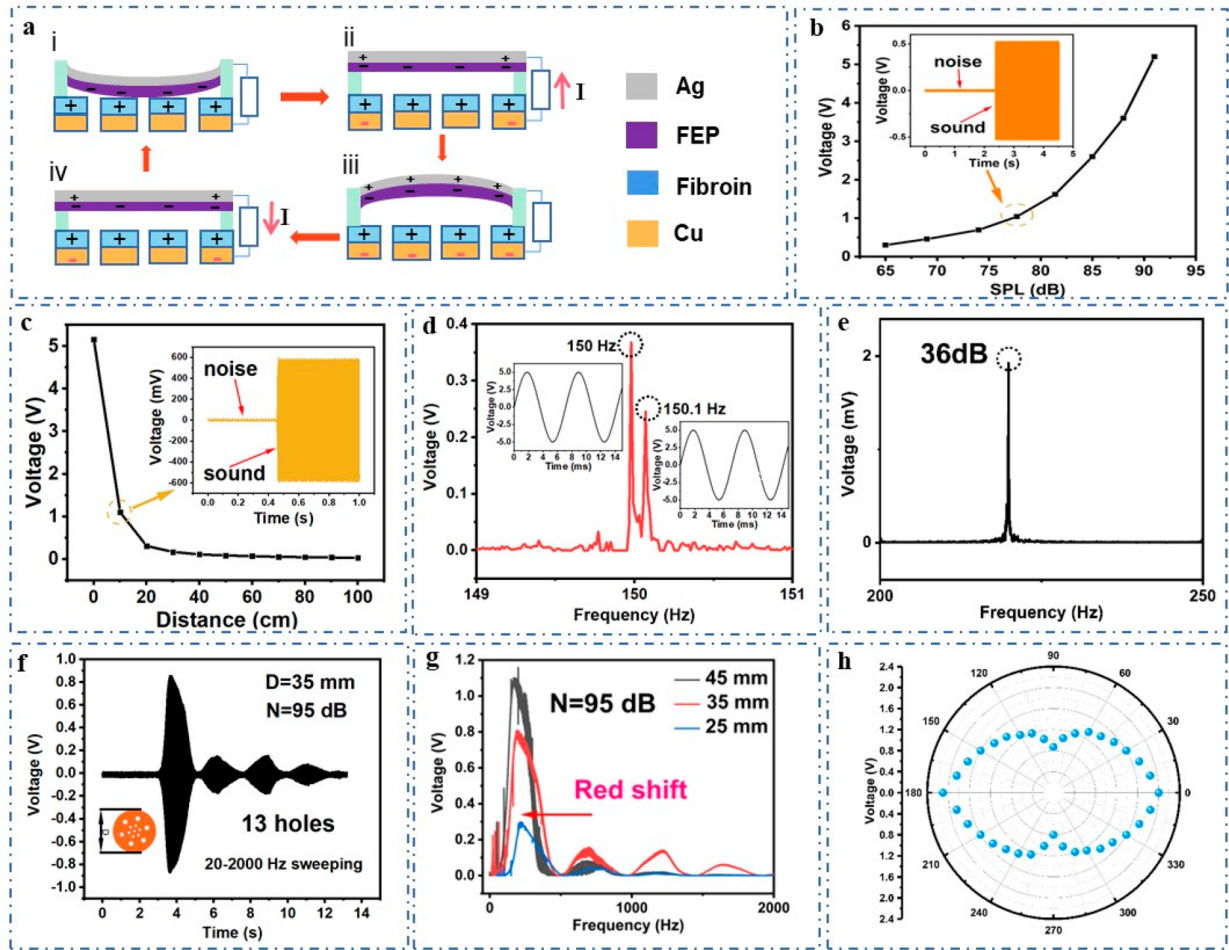
Working mechanism and acoustic performance and characterization of the VGST. (a) Schematic diagram of the working principle of the VGST under sound driven. (b) Effect of sound pressure level on the output voltage of the VGST and (c) effect of distances on the output voltage of the VGST. Inset shows the comparison of output voltage of the VGST caused by sound and environmental noise, respectively. (d) Corresponding FFT spectrum under sine wave excitation of 150 and 150.1 Hz. Inset shows the imposed sine wave excitation on the VGST. (e) Frequency spectrum of the VGST derived through Fourier transform when the sound intensity is 36 dB. (f) Voltage signal measured from the VGST under impact of sound source with sweeping frequency range of 20 to 2000 Hz and sound intensity of 95 dB. (g) The frequency spectra of the VGST derived through Fourier transform when diameter of the device varies. (h) Shape-dependent directional patterns of the VGST under sound intensity of 100 dB (*D* = 35 mm, where *D* is the diameter of the membrane).

The working principle of the VGST can be attributed to the coupling effect of contact electrification and electrostatic induction, as shown in Figure 2a and Note S1, where the inset figures are cross sections of TENG components. To precisely control the amplitude and frequency, Adobe Audition is chosen to provide a stable sine wave. As shown in Figure 2b, under the excitation of sine-wave sound of 150 Hz and keeping the sound source 2 cm away from the VGST, the output voltage of the VGST increases with increasing sound pressure level, and it can be calculated that a higher sensitivity of 167 mV/dB can be achieved. The results show that under higher sound intensity, the vibration between membranes is more intense, and the output voltage is higher, which can be expressed as the following equations:
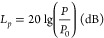
1
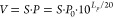
2where *V* is the peak output voltage, *S* is the sensitivity, *P* is the sound pressure, *P*_0_ is the reference sound pressure as 2 × 10^–5^ Pa, and *L*_*p*_ is the sound pressure. As can be seen from the inset figure in Figure 2c, at 77 dB, the electrical output signal is well distinguished from system noise. As shown in Figure 2c, keeping sound intensity at 90 dB, the output voltage drops from 5.2 V to nearly 0 V when the distance between the sound source and the VGST increases from 0 to 100 cm, indicating that the sound wave attenuates during propagation as the distance increases. When a 150 Hz sine-wave and a 150.1 Hz sine-wave are separately imposed on the VGST (Figure 2d), the peaks of the two frequencies can be clearly identified in the frequency domain through fast Fourier transformation, showing that the device has high accuracy to differentiate signals with a small frequency difference. When sine-wave sound source has a frequency of 150 Hz, a sound intensity of 36 dB is applied on the VGST. The output voltage is just larger than that of clutter signal, and its frequency peak can still be clearly seen through Fourier transform (Figure 2e), where 36 dB is almost close to the sound pressure level of quiet indoor environment, reflecting the good sensitivity of the fabricated VGST. Figure 2f shows the output voltage of the VGST (35 mm diameter, 95 dB sound intensity) under the impact of sound source with sweeping frequency range of 20–2000 Hz. It can be calculated from the setting time of sweeping frequency that when the sound frequency is 150 Hz, the voltage is maximum, which may be the resonant frequency of the VGST. Figure S3 shows that a 150 Hz sine-wave source is provided to VGST as an acoustic excitation. Since 150 Hz is the resonant frequency of the fabricated VGST device, maximum voltage output can be generated under the excitation of 150 Hz sound source. To further investigate the factors influencing the resonant frequency, we made VGSTs with different diameters of the membrane. Diameter is a key parameter affecting frequency response. Figure 2g shows that the VGST with a larger membrane diameter respond well to lower resonant frequency (red shift of frequency) with higher electric output. The results show that membranes with larger diameters can produce greater deformation and thus higher voltage output in response to the vibration of the acoustic wave, while the reduction in resonant frequency can be explained by the following equation:
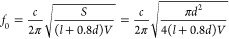
3where *f*_0_ is the resonant frequency of Helmholtz resonator, *c* is the speed of sound, *S* is the cross-sectional area of the neck or opening, *d* is the diameter of the neck or opening, *l* is the length of the neck, and *V* is the volume of the vessel.

We also tested the voltage signal of the VGST (35 mm diameter) at different decibels under impact of sound source in frequency range of 20–2000 Hz (Figure S4a–c) and found that their resonance frequencies were almost the same (Figure S4d), which is a good validation of the above equation. The resonance frequency is independent with the sound intensity. To evaluate the directional sound response characteristics, we detected the output voltage signal of the VGST in every direction. The experimental results show a butterfly-shaped directional pattern with mirror symmetry, as can be observed from Figure 2h. The response signal keeps a stable output in every direction, and reaches the maximum value at 0 and 180 deg and the minimum value at 90 and 270°, where 0° means the direction where sound source is perpendicular to the front view of the VGST. It can be seen that VGST responds in every direction, providing a broad-angle range for sound detection.

**Figure 3:**
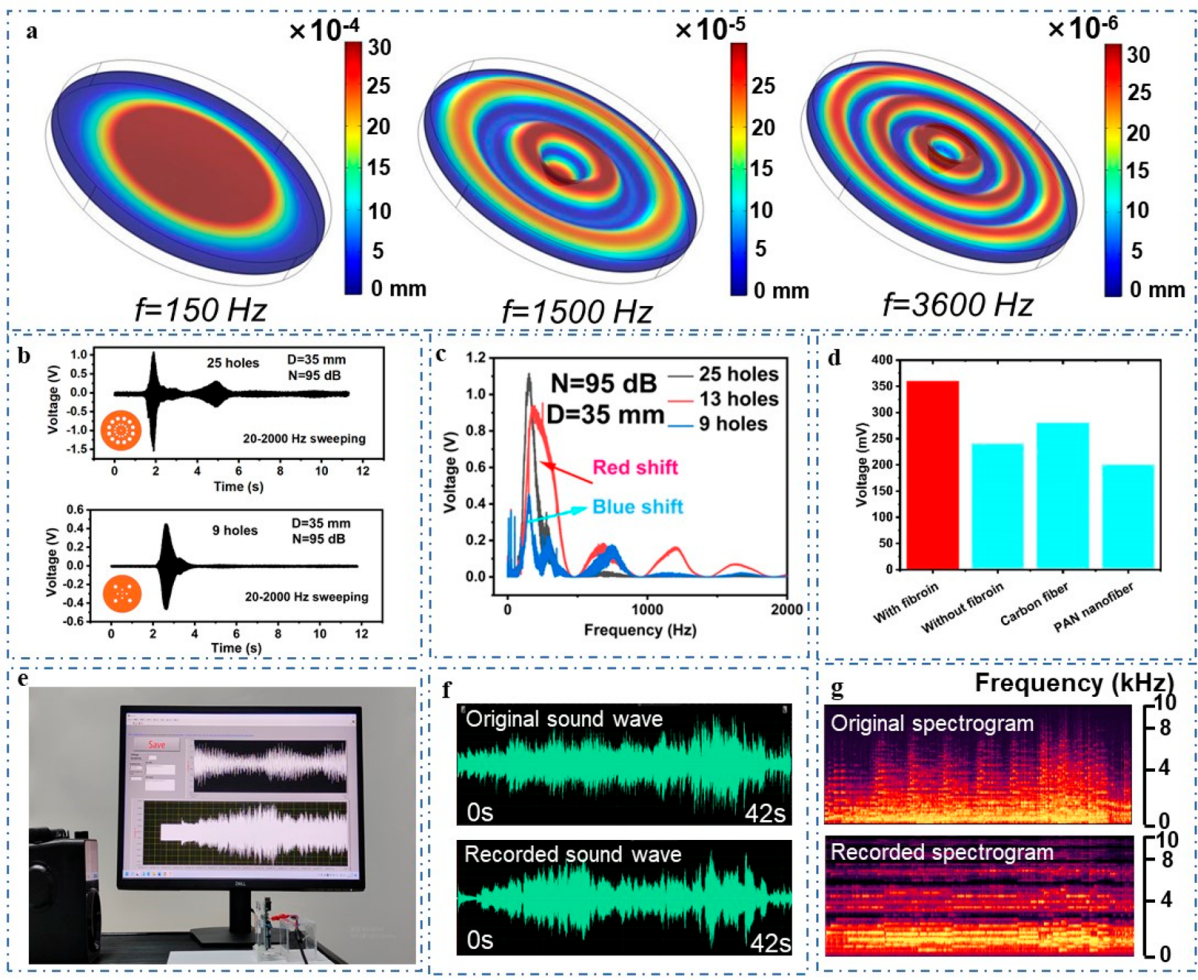
Other characteristics of the VGST and its application in recovering music. (a) Vibration patterns of FEP film under different frequencies (simulated using COMSOL under sound pressure of 1 Pa). (b) Output voltage measured by VGSTs with different number of holes (sweeping frequency range of 20–2000 Hz; sound intensity of 95 dB). (c) Frequency spectra of the VGST derived through Fourier transform when the number of holes of the VGST varies. (d) Comparison of output voltage of VGSTs with different electrification materials. (e) Application in recording the famous classical music “Blue Danube”. (f) Original music wave and recorded sound wave information and (g) their corresponding spectrograms.

In order to investigate the response of the VGST to sound source with different frequencies, finite element analysis method is adopted to simulate the deformation displacement of the circular film under three different acoustic frequencies. The modules and parameters of the finite element method are shown in Note S2. Usually, the first-order vibration mode provides the largest mechanical deformation of membrane, i.e., the largest deformation is under frequency of 150 Hz, while deformations under other frequencies are small, as shown in Figure 3a. Figure S5 shows the deformation displacements of the membrane at other frequencies, and Figure S6 shows deformation displacements under different sound intensities. Therefore, we mainly focused on modulating these modes of the VGST to broaden the sound frequency range with good response performance.

The vibration mode of the membrane conforms to Helmholtz and time harmonic equations:
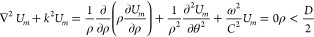
4
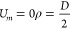
5where *U*_*m*_ is the envelope of the film, ρ is the polar radius, *k* represents the wavenumber (equal to ), ω is the angular frequency, *D* is the diameter of the film,  is the wave velocity, and *T* and σ represent the stretching force and surface mass density of the film, respectively.

In order to investigate what kind of membrane has the best acoustic response performance, we tested the performance of the VGSTs with different numbers of holes in the membrane (Figure 3b). Through frequency sweeping and Fourier transform, it can be found that when the membrane has 13 holes, the resonant frequency is the largest, and the resonant frequencies with membranes of 25 and 9 holes are almost the same (Figure 3c), while the output voltage of the VGST increases as the number of holes increases. The results show that the increase of hole numbers reduces the damping of the air to a greater extent, allowing the acoustic waves to drive membrane vibration better. Thus, the membrane displacement is increased, and the output voltage becomes larger. The resonant frequency is not determined by a certain factor, but it is determined by multiple factors. For example, the contact area between FEP and fibroin will decrease when the number of holes on the membrane is increased. Figure 3d depicts the performance comparison of the VGSTs with different triboelectric materials. It is easy to see that the output voltage is greater when copper is sprayed with silk protein than that without spraying. When the copper sheet is replaced by a carbon fiber mesh and PAN nanofiber films, the output voltage is smaller. By comparison, the effect of spraying silk protein on copper sheet is a better choice. This phenomenon is also due to the fact that silk protein is a very positive material and it generates a large amount of electron transfer upon contact with negative FEP film, resulting in a large output voltage. In addition, we have tested the stability of the VGST (Figure S7). A 90 dB, 220 Hz sine wave as an excitation source is supplied to the VGST every day, and it is can be seen from Figure S7, the output of the VGST remained stable even after 30 days. To demonstrate the high-fidelity of VGST, we used VGST to listen to a short section of the famous international classical music “Blue Danube” (Figure 3e) to generate an electrical signal. Then we used the software Adobe Audition 2020 to compare and analyze the original and recovered music. As shown in Figure 3f,g, the sound waves of the recovered music are a little different from those of the original music because we used wavelet denoising by MATLAB to remove the burr signal from the waveform (Figure S8) and converted the signal into a waveform audio file through MATLAB. Calculated by MATLAB, the mean square error (MSE) between the original and processed music is 1.0843^–4^, reflecting very high fidelity of the fabricated VGST. Video S1 demonstrates the process of listening and recovering music by VGST, showing its excellent sound recovery performance.

**Figure 4:**
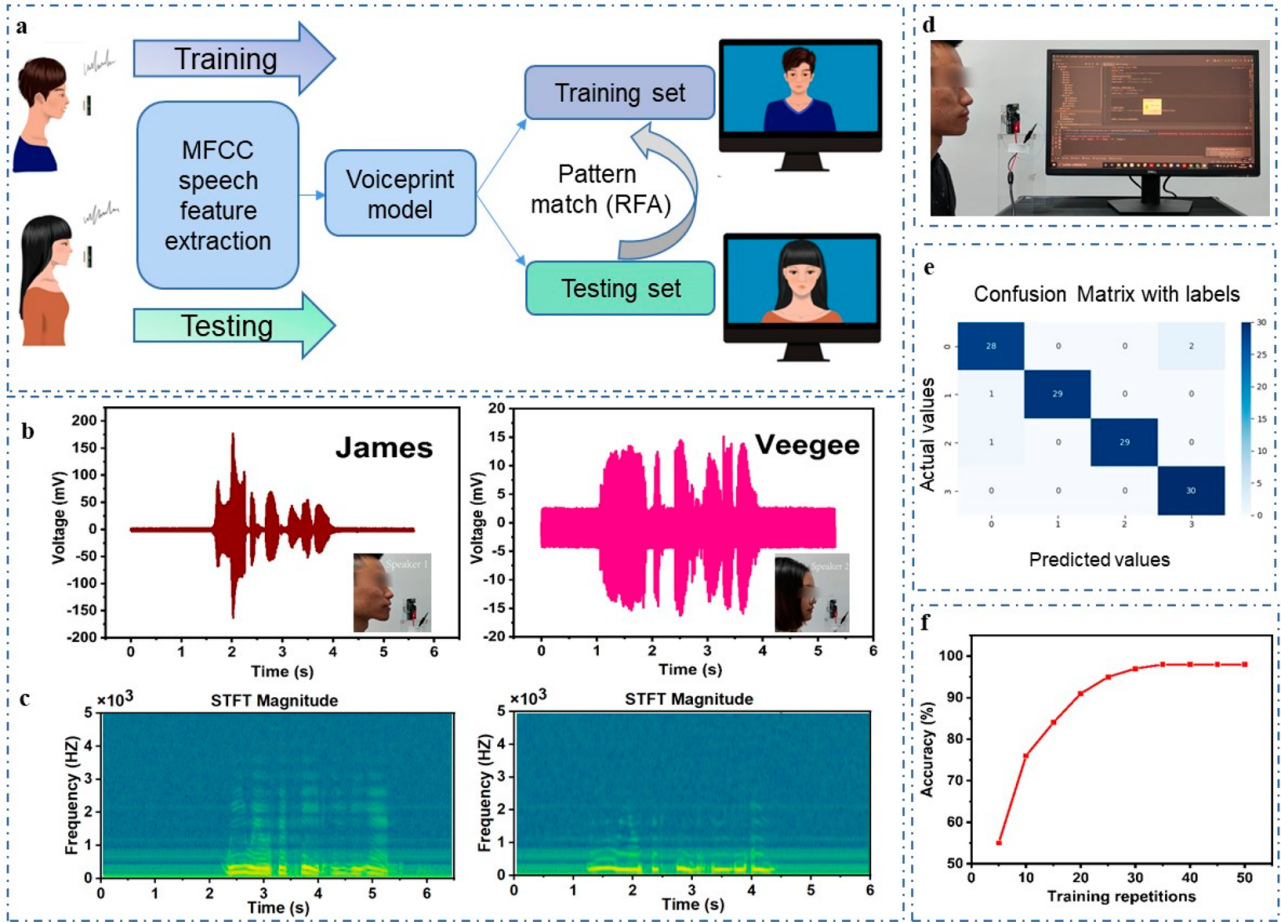
Investigation on VGST voice recognition system. (a) Voice recognition process for TENG-based speaker identification. (b) Output voltage of two people saying “We will succeed in the end.” (c) Corresponding STFT spectrograms of the acquired sound waveforms for the two people. (d) Demonstration of the TENG-based voice recognition system. (e) Confusion matrix diagram for machine learning outcome. (f) Overall accuracy of voice recognition at different training repetitions using random forest algorithm.

Information security construction is an indispensable part of e-government construction and national security system. Password-based authentication is one of the most commonly used ways to consolidate information security in today’s society,^48^ including voice password. As an important kind of biometric feature, the human voice usually contains rich information, and the collection of human voice is very convenient, so the analysis of human voice signals has significant application values. Figure 4a depicts the overall process for voice recognition of VGST. First, two men and two women were selected as volunteers, each of whom speaks 100 sentences. We extract the eigenvalues of the sentences by calculating the Mel Frequency Cepstrum Coefficient (MFCC) to build a database. The specific process for obtaining MFCC features is described in Note S3. Then 70 sentences out of 100 sentences are selected as the training set, and 30 sentences are selected as the testing set. As a new machine learning model, a random forest algorithm is used to establish a voice pattern model, which has high accuracy, good noise immunity, fast training speed, small calculation volume, and the ability to run effectively on large data sets. When the testing set is matched with the training set, it can judge which speaker each person’s speech signal comes from. The principle of the algorithm is simple which consists of a large amount of decision trees. When a new sample comes in, each decision tree in the forest makes a separate determination to see which category the sample should belong to. The phrase “We will succeed in the end” was chosen as the test statement in our experiment. Each person used this phrase to speak to the TENG, and the computer interface was able to display the speaker’s name immediately. Figure 4b shows the output voltage waveforms of the voice signals of James and Veegee, and the corresponding information on their voice signals in frequency domain is shown in Figure 4c. The output voltage waveform and frequency domain information on the other two volunteers are reflected in the Figure S9. It has been observed that the frequency of a normal human voice is between 50 and 500 Hz, but each person’s voice frequency corresponds to a different amplitude. By following the frequency information on each person, the VGST is able to distinguish their signals very well. In the voiceprint recognition system, the experimenter interacts with the VGST (Figure 4d and Video S2). The reliability of the VGST in voiceprint recognition was demonstrated in Figure 4e, which is the confusion matrix plot, reflecting the recognition accuracy of 97%. Figure 4f displays that when training is repeated 5 times, the accuracy rate reaches 55%. As the number of training repetitions increases, the accuracy rate will increase and finally can reach 97%. We tested it using XGB algorithm compared with the random forest algorithm. Figure S10 are the learning curves calculated through both algorithms. It is not difficult to find that the accuracy of random forest algorithm is higher.

**Figure 5:**
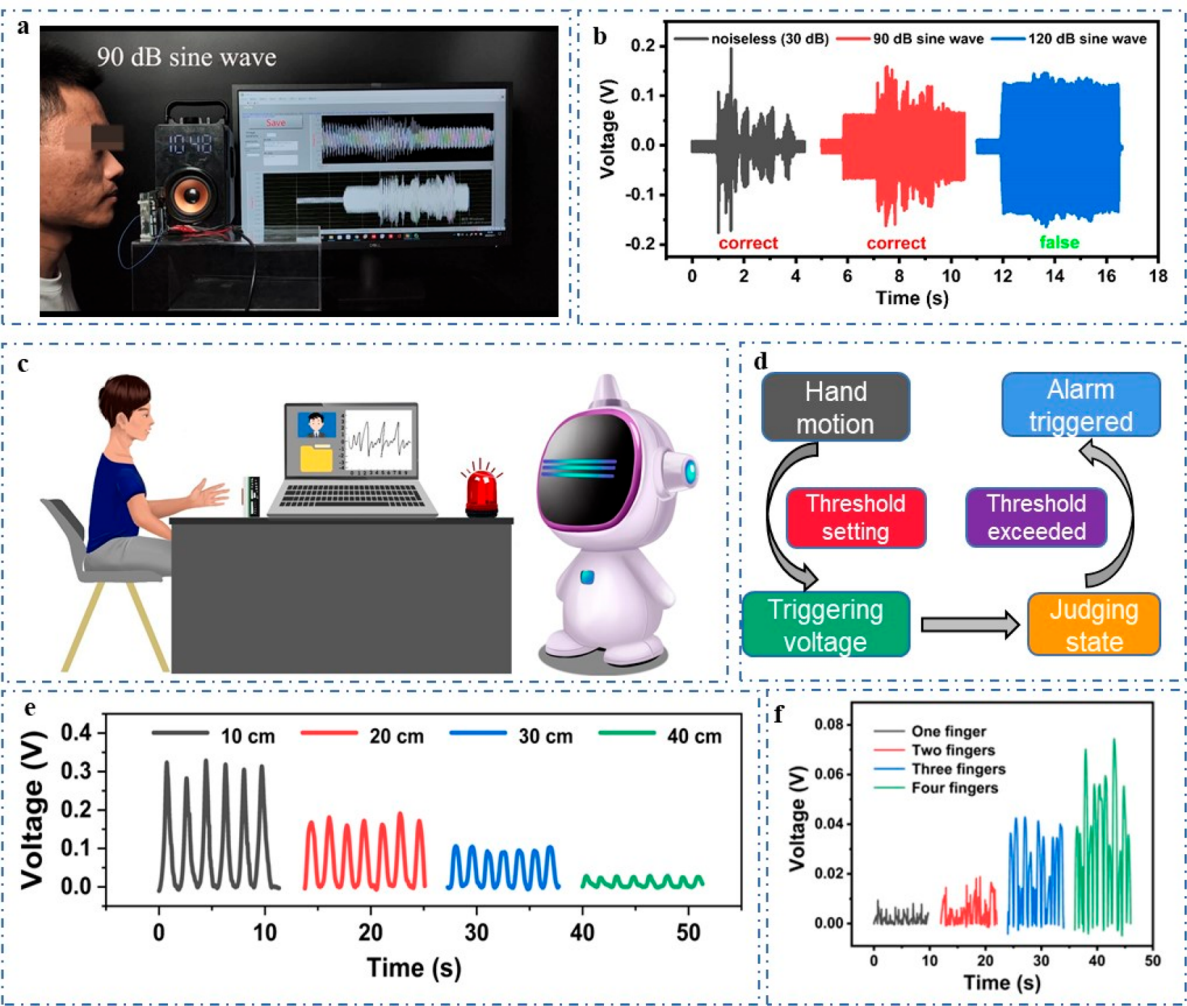
The VGST’s antinoise performance and applications in gesture recognition. (a) Demonstration of the VGST’s antinoise performance. (b) Speech waveforms at different noise intensity. (c) Application scenarios of gesture recognition. (d) Working flow of gesture recognition. (e) Output voltage corresponding to four different distances between hand and the device. (f) Output voltage corresponding to four different gestures.

In order to test the antinoise performance of the VGST, we used an electronic speaker to create a 220 Hz sine wave as noise source. The noise source is 2 cm away from the device and it is vertical to the front side of the membrane. The electronic speaker released noise when the volunteer spoke to the VGST, as shown in Figure 5a and Video S3. In Figure 5b, human voice can be clearly distinguished in computer interface in a quiet environment and the human identity can be recognized accurately. In a noisy environment under 90 dB, the human voice can still be easily distinguished and the recognition is still correct. Human voice signals can be recognized correctly until the noise intensity continues to increase to 120 dB. The human voice waveform is almost drowned by the noise under sound intensity of 120 dB, where the recognition starts to be wrong, demonstrating the good antinoise performance of the VGST. Except for its application on voice recognition, the VGST can also be applied to gesture recognition. Human–machine interaction based on human hand movement is one of the most important methods and here we use the principle of noncontact electrostatic induction to judge the state of hand movement. The real-world demonstration of gesture recognition is shown in Figure 5c, and the circuit modules used are shown in Figure S11. The functions of the development board are described in Note S4. The whole process can be divided into four parts (Figure 5d). Once the threshold is set, the hand moves to generate an electrostatic induction signal and to trigger the alarm system. Then we can judge the hand state according to the output voltage generated from the device and displayed on the computer. If the voltage exceeds the set threshold, the alarm can be triggered to realize a warning effect. According to this working principle, the distance between the hand and the device can be judged according to the output voltage of the VGST (Figure 5e). In the initial state, the hand is kept directly in front of the VGST with a vertical distance of 50 cm, and there is no output signal. When the hand slowly moves to only 10 cm away from the VGST, the induced voltage is 0.32 V. With the decrease of the distance between hand and device, the induced output voltage becomes larger. A linear relationship between the output voltage and distance can be found, thus the distance between hand and the VGST can be judged by the output voltage. Next, we demonstrate four hand-gesture recognitions from 1 to 4. At the beginning, we keep the hand in the upper position before the VGST, and each gesture moves down slowly until the fingers completely pass by the VGST. According to Figure 5f, the more fingers in the gesture, the greater the generated output voltage signal. Therefore, the gesture can be judged according to the output voltage of the VGST. From this work, it can be seen that gesture recognition based on the principle of electrostatic induction has a good prospect, and it could be prospected that more complex gestures can be recognized by means of machine learning and three-dimensional spatial positioning.

## 3 Conclusions

In summary, we have designed and developed a thin triboelectric translator called a VGST that can translate the raw signals into electrical signals and realize both voice and gesture recognition with high sensitivity. By spraying silk protein on the copper of the device, it can detect voice in frequency range of 20–2000 Hz with resolution of 0.1 Hz and sensitivity of 167 mV/dB. By changing the diameter and holes of the device, the frequency response of the VGST device can be changed and modified. This fabricated VGST can act as a high-fidelity listening platform, effectively recording and restoring sound signals. In addition, with the aid of machine learning, the VGST can recognize voice information with high accuracy of 97% under less than 120 dB noisy environment and has good antinoise performance. It can also be used for gesture recognition, judging the distance between human hand and the VGST device and recognizing different gestures. It can be seen that the demonstrated VGST with multifunctions can enrich the methods of human–machine interaction and has huge potential applications in many areas such as authentication, information security, high-fidelity platforms, machine control, and medical rehabilitation et al.

## 4 Experimental Section

### 4.1 Fabrication of VGST

First, an acrylic board with a thickness of 2 mm was cut into a circular substrate (diameter of 35 mm) by a laser cutting machine (LM-1390), and some holes were pierced on the circular substrate according to the preset sizes. The 60 μm thick copper sheet was attached to the perforated acrylic substrate as the upper electrode, then a hobby knife was used to dig some holes as designed on the acrylic substrate to reduce air damping. 0.2 g of fibroin powder was dissolved in water to form a 15% solution (13.35 mL), then stirred for 5 min until uniform using a magnetic stirring apparatus. Later, the stirred solution was poured into a spray gun and sprayed evenly on the surface of the copper sheet until a white film is formed. The protein film was left for 20 min at room temperature of 20 °C, which can be used as an electrification layer. Then a layer of 80 nm silver was coated on the 12.5 um FEP film as the lower electrode using a magnetron sputtering machine. A polarizer was used for 10 min to provide a high voltage of 6 kV and some negative charges were injected on the surface of FEP. Three circular-ring paper sheets (each 0.1 mm thick) were used as spacers to separate the top and bottom membranes.

### 4.2 Integration of VGST with Development Board

An acrylic board was cut into a rectangle (6.75 cm long and 5.25 cm wide) and was cut with certain circular holes with a diameter of 3.3 cm in the center. Then glue was applied around the holes so that the VGST can be attached to the acrylic board. The four corners of the rectangular acrylic board were punched with holes, using nuts to integrate the VGST with development board.

### 4.3 Characterization and Measurement

SU8020 cold field scanning electron microscope was used to characterize the surface morphology of fibroin membrane as well as FEP membrane. Adobe Audition was used to provide stable sine wave with adjustable frequency. Norwest H3 Bluetooth speaker was used to drive the membrane vibration of the VGST. Voltage preamplifier (Stanford SR560) was used to measure the voltage signal of sound. Programmable electrostatic meter (Keithley 6514) was used to measure the gesture of voltage signal. Acquisition card NI USB-6356 was used for data acquisition. Software platform was built based on Labview for real-time data presentation. Analysis and voice recognition interface was built with Python. The *sklearn* library was used for machine learning training and testing.

## Data Availability

The data that support the findings of this study are available for the corresponding author upon reasonable request.

## Supporting Information Available

Views of the VGST; output voltage and frequency spectra; COMSOL deformation of films; stability test of the VGST; spectrograms of music and wavelet denoising by MATLAB; output voltage waveforms and the spectra of two other volunteers; illustration of the gesture recognition function of the VGST; electronic module; working mechanism of the VGST; simulation parameters using finite element method; specific process for obtaining MFCC features; functions of development board (PDF)
Video S1: Recording the famous classical music (MP4)
Video S2: Voice recognition process in demonstration of TENG-based speaker identification (MP4)
Video S3: Test of VGST antinoise performance (MP4)
