# Supplementary material for: Human–Machine Interaction via Dual Modes of Voice and Gesture Enabled by Triboelectric Nanogenerator and Machine Learning
Source: ACS Appl Mater Interfaces. 2023 Mar 22;15(13):17009–18. doi: 10.1021/acsami.3c00566 (PMC10080540; doi:10.1021/acsami.3c00566)
Supplement: Supplementary file 1 — am3c00566_si_001.pdf [file am3c00566_si_001.pdf]

Supporting Information

Human-Machine Interaction via Dual Modes of Voice and Gesture Enabled by Triboelectric Nanogenerator and Machine Learning

Hao Luo [a,b], Jingyi Du [a,b], Peng Yang[b,c], Yuxiang Shi[b,c], Zhaoqi Liu[b,c], Dehong Yang[b,c], Li Zheng [a]*, Xiangyu Chen [b,c]*, Zhong Lin Wang [b,c]*

[a]*College of mathematics and physics, Shanghai Key Laboratory of Materials Protection and Advanced Materials in Electric Power, Shanghai University of Electric Power, Shanghai 200090, China*

[b]*Beijing Key Laboratory of Micro-nano Energy and Sensor, Beijing Institute of Nanoenergy and Nanosystems, Chinese Academy of Sciences Beijing 100083, P. R. China*

[c] *School of Nanoscience and Technology, University of Chinese Academy of Sciences Beijing 100049, P. R. China*

*** To whom correspondence should be addressed: zhengli@shiep.edu.cn, chenxiangyu@binn.cas.cn, zhong.wang@mse.gatech.edu**

Note S1: Working mechanism of VGST. When acoustic wave induces membrane deformation, the membrane vibration causes electric signal output. When there exists an acoustic source, the generated air pressure drives the FEP membrane to vibrate, and thus generate an electrical output signal. Following is the exact process. When FEP and fibroin membrane are in full contact, the surfaces of the two films carry equal opposite charges, as shown in Figure 2a-i. Since these charges are only confined on the surface, and the two kinds of charges are in the same plane, there is no potential difference between two corresponding electrodes. When the two films gradually separate, an electrostatic potential will be generated between Cu and Ag electrodes, resulting in an instantaneous current from Cu to Ag (Figure 2a-ii). The flow of electrons lasts until the separation of the two contact surfaces is maximized (Figure 2a-iii). Thereafter, the FEP film is pushed back toward the fibroin film by the action of the sound wave. Free electrons in the Cu film flow back to the conductive Ag electrode through the external circuit (Figure 2a-iv). A full circle will be completed after the FEP comes back to the bottom electrode (Figure 2ai).

Note S2: We use the coupling of the acoustic frequency domain interface and the solid mechanical interface to simulate the vibration mode of the fabricated VGST device in different frequency domain. Three-dimensional space is taken as the test case. Two vertically arranged cylinders with

radius of 10 mm and height of 1 mm are used as sound fields. A cylinder with radius of 10 mm and thickness of 0.1 mm representing the film in VGST device is located between the two vertically arranged cylinders, and the sound transmission medium is set as air.

Note S3: Extracting MFCC features is roughly done by pre-emphasis, framing, windowing, fast Fourier transform, filtering by Mel filter bank, taking logarithm and discrete cosine transform. When a person makes a voice, the high-frequency part of the voice signal will be weakened due to the action of the vocal cords and lips and teeth. Pre-emphasis is used to enhance the high frequency band by using a first-order digital high-pass filter to make the spectrum more balanced. Framing and windowing mainly divide the voice signal into N frames to make each frame approximately smooth and reduce the spectral leakage. Then the time domain signal is converted to frequency domain by fast Fourier transform, and then converted to a frequency more suitable for human ear by Mel filtering. By taking the logarithm and discrete cosine transform (DCT), the coupling relationship between the dimensions of the features is removed, and the MFCC features are obtained.

Note S4: The development board we used is the Arduino development board. Arduino is an open-source platform that is easy to learn and has multiple functions, which consists of two parts: hardware and software (Arduino IDE). The Arduino hardware part can work independently, and can also be used in conjunction with external hardware devices. For example, we can use Arduino combined with various sensors to sense the environment, use Arduino control motor to drive robotic arms, robots and drones, and use Arduino to control colorful LED. Arduino IDE software is the program development environment of Arduino development board. We write our program code in the IDE and upload it to the Arduino, and the Arduino could do what we tell it to do.

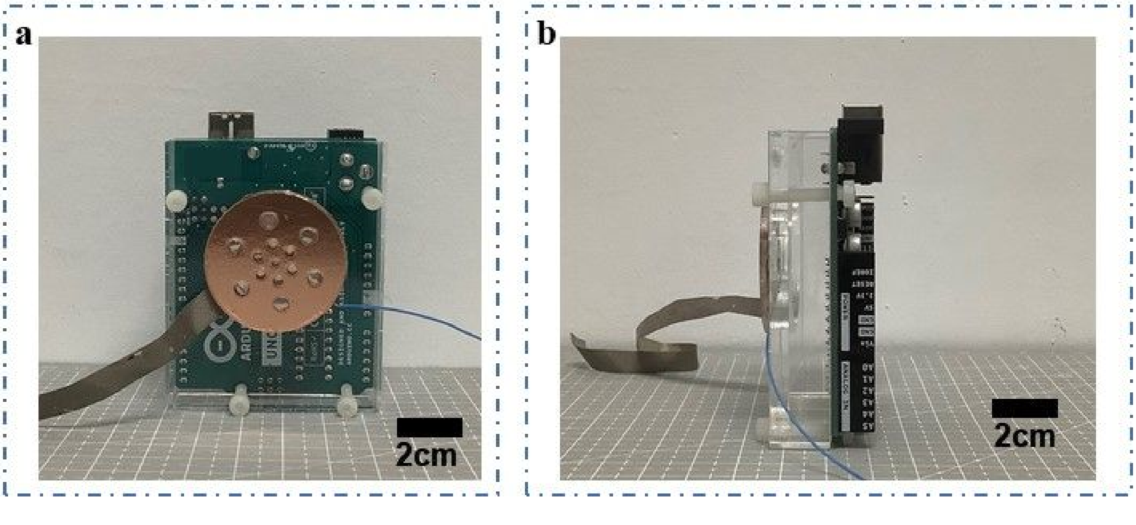

Figure S1. (a) Front view and (b) side view of the fabricated VGST device.

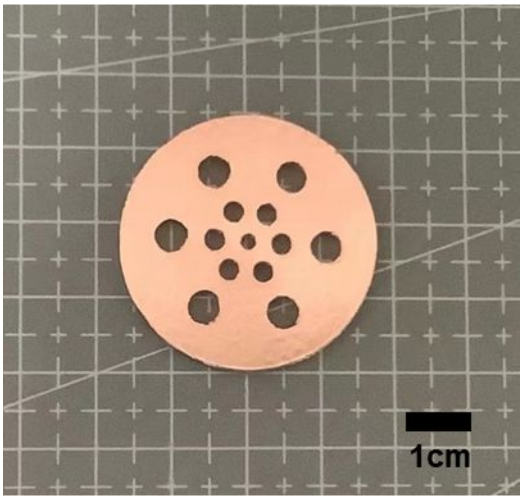

Figure S2. The picture of perforated copper sheet coated with silk protein.

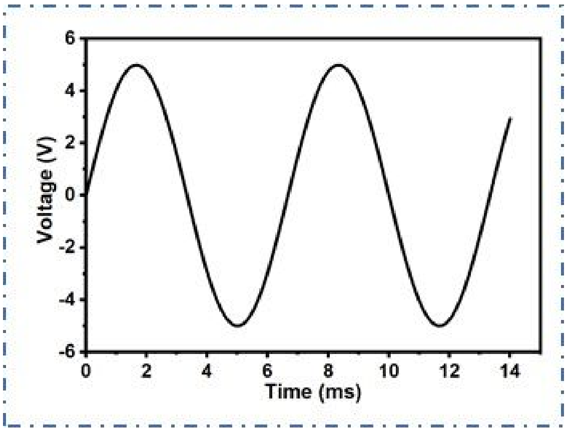

Figure S3. A sine-wave excitation of 150 Hz is applied to the VGST.

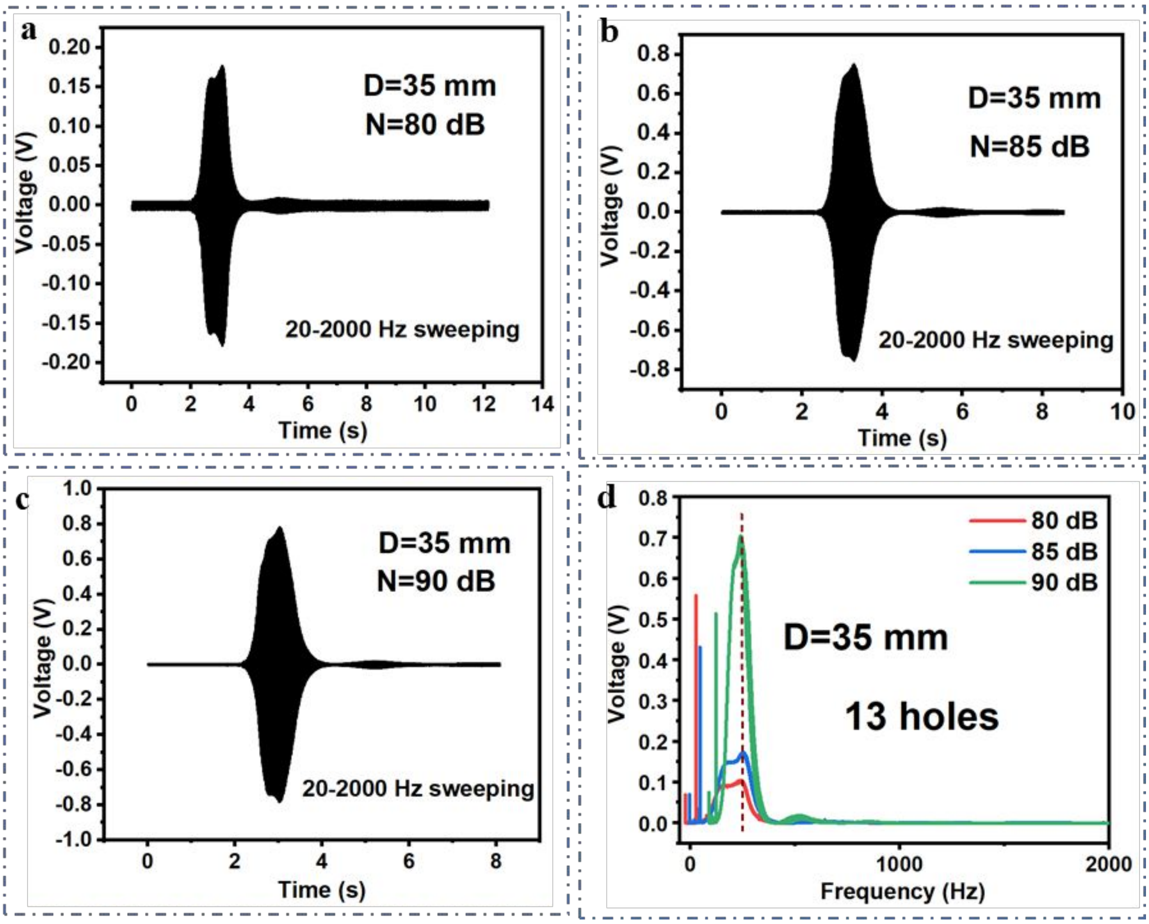

Figure S4. Output voltage measured by VGSTs at (a) 80 dB, (b) 85 dB, (c) 90 dB (sweeping frequency range of 20 to 2000 Hz). (d) Frequency spectrums of TENG derived through Fourier transform under different sound intensities.

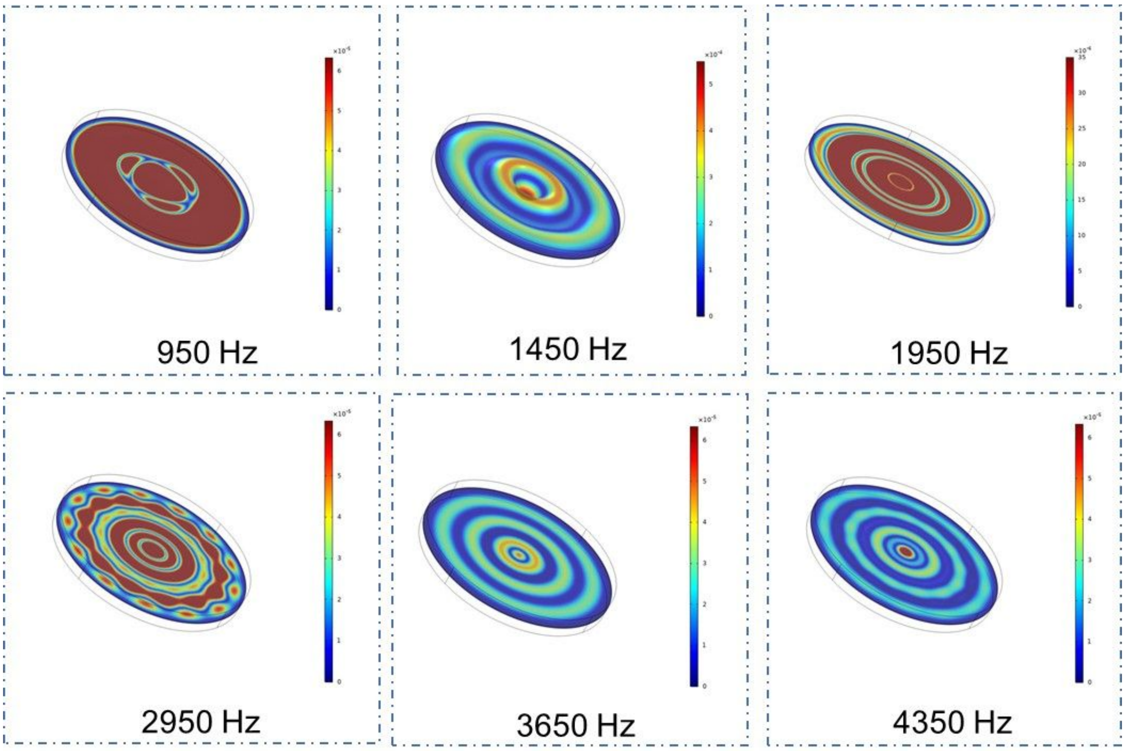

Figure S5. Vibration patterns of FEP film under other different frequencies (simulated using COMSOL under sound pressure of 1 Pa).

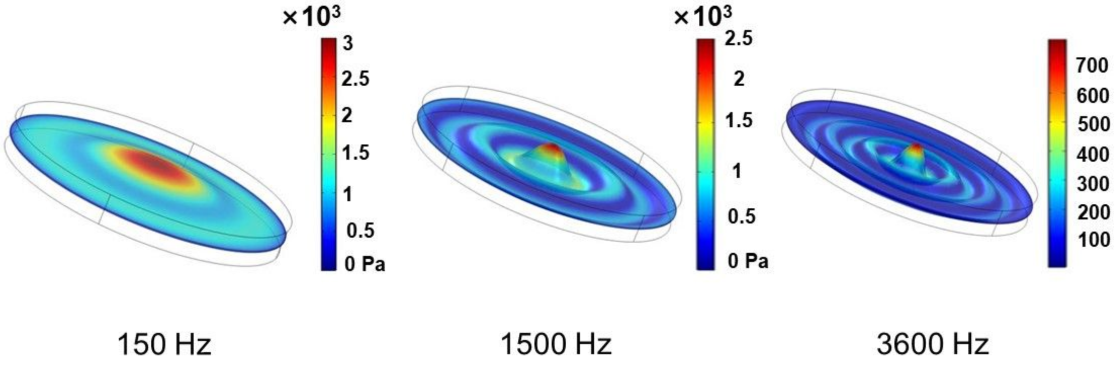

Figure S6. COMSOL simulation on deformation of membranes under different sound intensities.

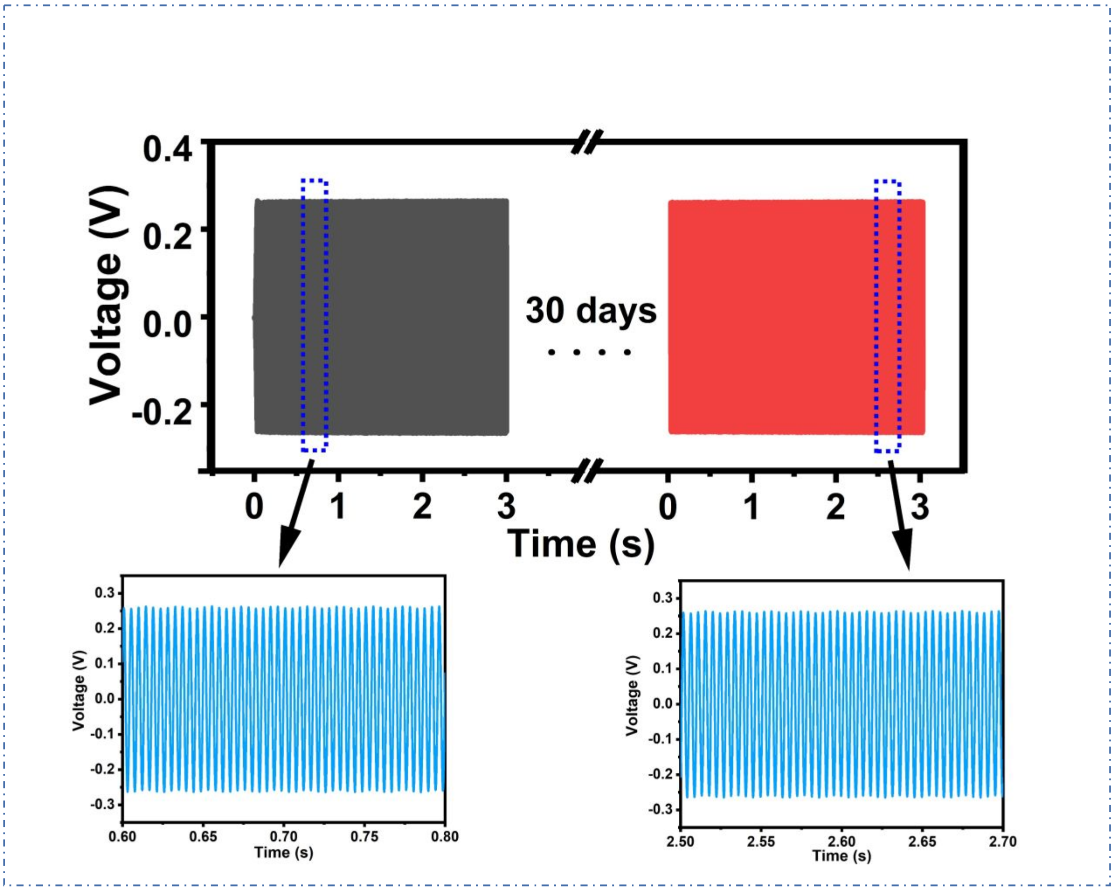

Figure S7. Stability test of VGST during 30 days continuous stimulation under a frequency of 220 Hz and SPL of 90 dB.

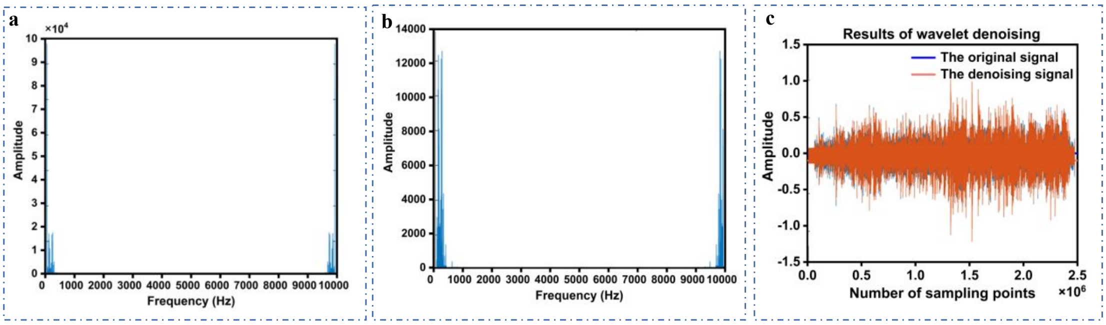

Figure S8. Spectrograms of music (a) before and (b) after filtering. (c) Wavelet denoising by MATLAB.

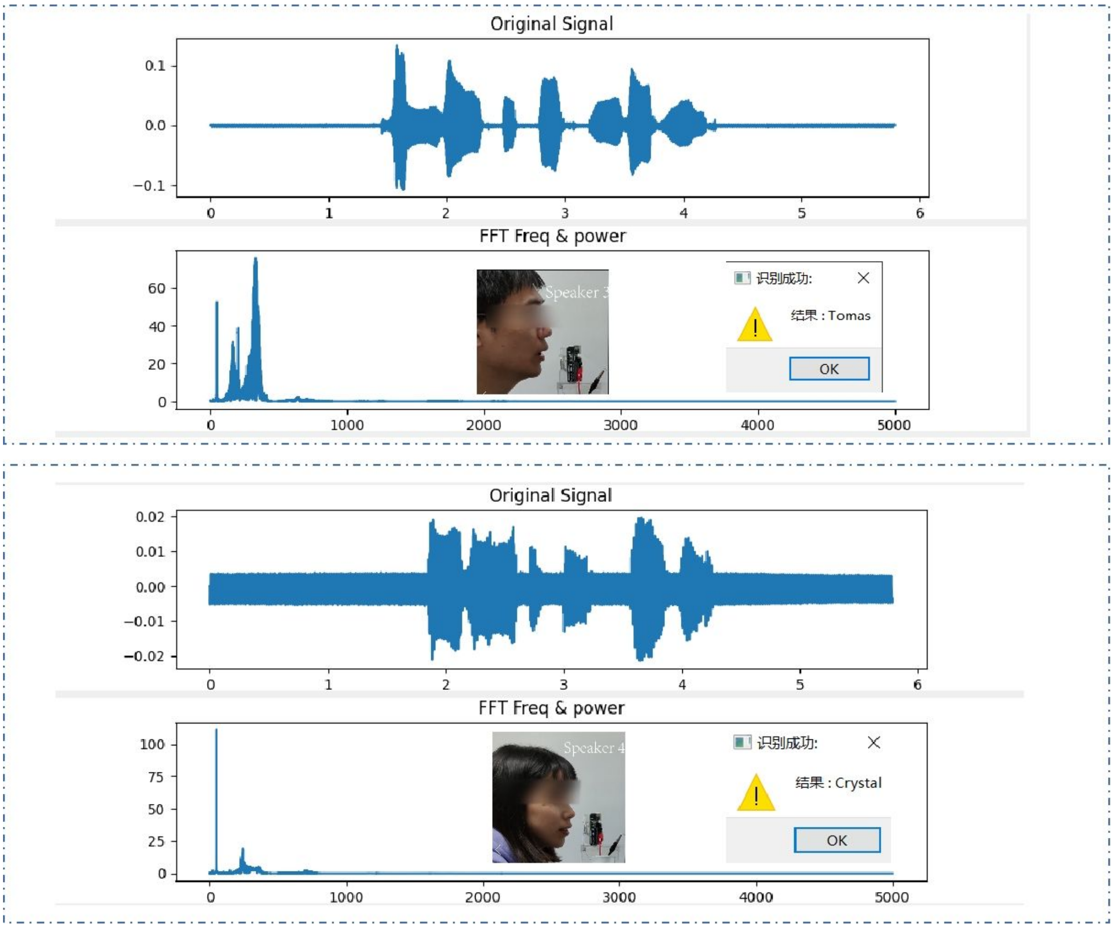

Figure S9. Output voltage waveforms of VGST and the spectrums of two volunteers saying "We will succeed in the end."

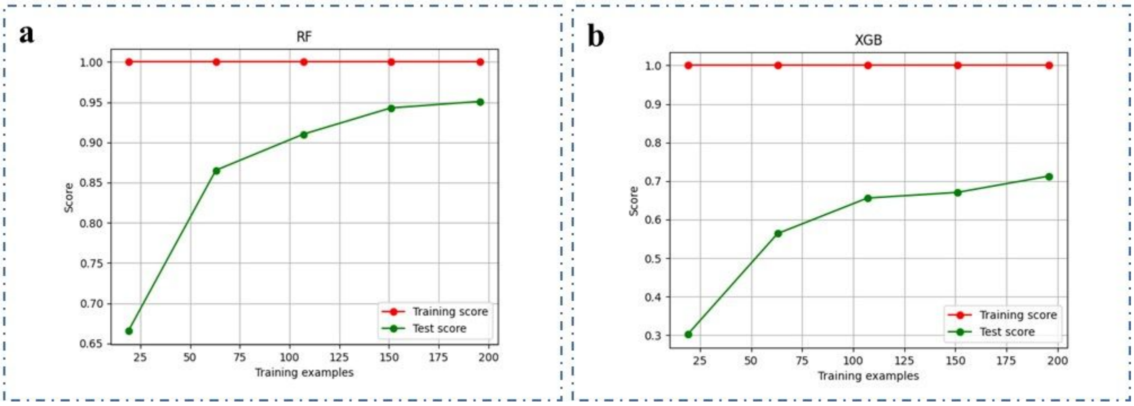

Figure S10. The learning curves through (a) random forest algorithm and (b) XGB algorithm.

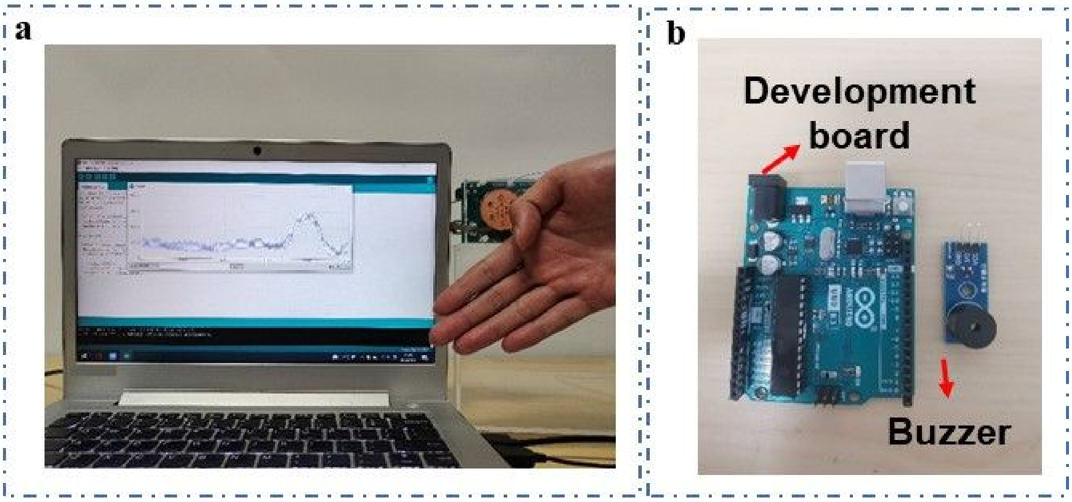

Figure S11. (a) Illustration of the gesture recognition function of the VGST. (b) Electronic module used for potential application demonstration.
